# Interleukin-6 deficiency modulates testicular function by increasing the expression of suppressor of cytokine signaling 3 (SOCS3) in mice

**DOI:** 10.1038/s41598-021-90872-6

**Published:** 2021-06-01

**Authors:** Thaís Alves-Silva, Geanne Arantes Freitas, Talita Guerreiro Rodrigues Húngaro, Adriano Cleis Arruda, Lila Missae Oyama, Maria Christina Werneck Avellar, Ronaldo Carvalho Araujo

**Affiliations:** 1 Laboratory of Genetics and Exercise Metabolism, Biophysics Department, Federal University of São Paulo (UNIFESP), São Paulo, Brazil; 2 Molecular Biology Program, Federal University of São Paulo (UNIFESP), São Paulo, Brazil; 3 Pharmacology and Molecular Biology Institute, Federal University of São Paulo (UNIFESP), São Paulo, Brazil; 4 Institute of Biomedical Sciences, University of São Paulo (USP), São Paulo, Brazil; 5 Nephrology Program, Federal University of São Paulo (UNIFESP), São Paulo, Brazil; 6 Laboratory of Nutrition and Endocrine Physiology, Physiology Department, Federal University of São Paulo (UNIFESP), São Paulo, Brazil

**Keywords:** Cytokines, Reproductive biology

## Abstract

Several cytokines have been reported to participate in spermatogenesis, including interleukin-6 (IL6). However, not many studies have been conducted on the loss of *Il6* on the male reproductive tract. Nonetheless, there is considerable knowledge regarding the pathological and physiological role of IL6 on spermatogenesis. In this way, this study evaluated the impact of *Il6* deficiency on mice testicles in the absence of infection or inflammation. We showed that *Il6* deficiency increases daily sperm production, the number of spermatids, and the testicular testosterone and dihydrotestosterone levels. Besides that, mice with a deleted *Il6* (IL6KO) showed increased testicular SOCS3 levels, with no changes in pJAK/JAK and pSTAT3/STAT3 ratios. It is worth noting that the aforementioned pathway is not the only pathway to up-regulate SOCS3, nor is it the only SOCS3 target, thus proposing that the increase of SOCS3 in the testis occurs independently of the JAK-STAT signaling in IL6KO mice. Therefore, we suggest that the lack of *Il6* drives androgenic production by increasing SOCS3 in the testis, thus leading to an increase in spermatogenesis.

## Introduction

Spermatogenesis is the physiological process by which male germ cell precursors develop into sperm cells. This process occurs in the seminiferous epithelium of adult testis, which is composed of Sertoli cells and spermatogenic cells. Several cytokines, such as transforming growth factor, beta 1 (TGFB1), interleukin-6 (IL6), interferon gamma (IFNG), and tumor necrosis factor (TNF) have been described to participate in this process^1–3^.

IL6 is a pleiotropic cytokine secreted by many cell types^4–6^. Despite the variety of cells producing IL6, it acts mainly via one membrane receptor, the IL6 receptor, alpha (IL6R)^7,8^, which is expressed only in certain cell types, including the Sertoli cells, Leydig cells, and spermatogenic cells^3,9–11^. In addition to classic IL6 signaling (through membrane receptor), there is an alternative signaling or trans-signaling, which takes place through the soluble IL6 receptor (sIL6R). Glycoprotein 130 (GP130) is the signal transductor of the IL6 pathway, which heterodimerizes when it binds to the IL6-(s)IL6R complex^6–8^. The biochemical interaction between GP130 and the receptor activates the following pathways: Janus kinase—signal transducer and activator transcription—suppressor of cytokine signaling 3 (JAK-STAT-SOCS3), extracellular signal-regulated kinases / mitogen-activated protein kinase (ERK/MAPK), and phosphoinositide-3-kinase—protein kinase B (PI3K-AKT)^12–14^.

Some authors have already demonstrated that IL6 is likely to have both a pathological and a physiological role in the testis and in the epididymis, where the spermatozoa acquire motility and the ability to fertilize the egg^3,15–18^. Furthermore, the transcription factor STAT3 is relevant for spermatogonia and spermatogonial stem cell differentiation^19,20^. Moreover, sperm head defects and teratozoospermia indices are negatively related to phosphorylated STAT3 levels in humans^21^. However, not many studies on *Il6* deficiency on the male reproductive tract have been conducted, which further reinforces the need to elucidate the role of IL6 on spermatogenesis.

Systemic or testicular injuries, such as intraperitoneal bacterial lipopolysaccharides (LPS) injection and autoimmune orchitis, respectively, increase testicular IL6 levels^2,15,22–25^. In vitro experiments have shown that IL6 disturbs the blood-testis barrier^24–26^, inhibits the meiotic DNA synthesis in pre-leptotene spermatocytes^27^, influences the permeability of Sertoli cells' tight junction^24^, affects the secretion of transferrin and inhibin B by Sertoli cells^28,29^, reduces sperm motility^30,31^, and suppresses testosterone secretion by Leydig cells^32^. Consequently, the overexpression of IL6 has been shown to impair spermatogenesis and fertility.

Knockout mice models are used to understand the role of proteins in vital processes. In respect to *Il6* knockout (IL6KO) mice, although they appear to have a normal testicular function, since they are good breeders^33^, there are no studies about their reproductive tract under physiological conditions. However, IL6KO mice do not exhibit the feminization phenotype caused by the chronic cysticercosis observed in wild-type (WT) mice, which is characterized by low testosterone and high estradiol levels^17^.

Given the above, our hypothesis is that *Il6* deficiency interferes with androgen production by up-regulating it, which can intensify sperm production. Given the limited literature exploring the male reproductive tract of IL6KO mice and the evidence corroborating our hypothesis, the study aims to investigate the effects of *Il6* deficiency on testicular function in the absence of infection or inflammation, as well as to determine whether the loss of this cytokine changes the testicular JAK-STAT pathway.

## Results

### IL6KO mice are leaner, have reduced epididymal adipose tissue, and increased relative testis weight

After confirming that the IL6KO group had no functional IL6 (Supplementary Result 1), we observed that *Il6* deficiency has altered the body composition of these animals compared to WT mice. IL6KO mice are leaner than WT (*p* = 0.0118), have reduced epididymal adipose tissue (*p* = 0.0042), increased relative testis weight (*p* = 0.0344), and a trend towards increased relative epididymis weight (*p* = 0.0667), with no changes on the absolute mass of these organs, nor on the other tissues analyzed (Table 1).

**Table 1:** Body weight and absolute and relative reproductive and metabolic organs weight.

	Body weight (g)	Testis (mg)	Epididymis (mg)	Epididymal Adipose Tissue (mg)	Muscle (mg)	Liver (mg)
**Absolute**						
WT (n = 12)	27.43 ± 0.51	90.5 ± 4	36.8 ± 2 (n = 6)	384.3 ± 24	129.5 ± 6	1360 ± 57
IL6KO (n = 9)	25.06 ± 0.63 *	99.7 ± 4	40.5 ± 3 (n = 5)	286.8 ± 10 *	122.5 ± 7	1259 ± 23
**Relative**^**1**^						
WT (n = 12)		3.31 ± 0.16	1.32 ± 0.05 (n = 6)	14.27 ± 0.88	4.80 ± 0.20	50.55 ± 2.20
IL6KO (n = 9)		3.96 ± 0.25 *	1.49 ± 0.04§ (n = 5)	11.88 ± 0.48 *	5.07 ± 0.30	52.02 ± 0.46

^**1**^ Relative weight = organ weight (mg)/body weight (g).

*p < 0.05, ^§^*p* = 0.067, unpaired *t*-test or Mann–Whitney test, GraphPad Prism 6. Values expressed as mean ± SEM.

### *Il6* deficiency alters sperm production, accompanied by testicular hormonal changes

In order to analyze the impacts of *Il6* absence on the male reproductive tract, we determined the daily sperm production (DSP), epididymal sperm transit time, and sperm count, as quantitative sperm parameters, histologic and morphometric analyses, and sex hormone levels measurements.

#### Increased daily sperm production and testicular spermatids, in addition to a thicker layer of seminiferous epithelium on IL6KO mice

**Figure 1:**
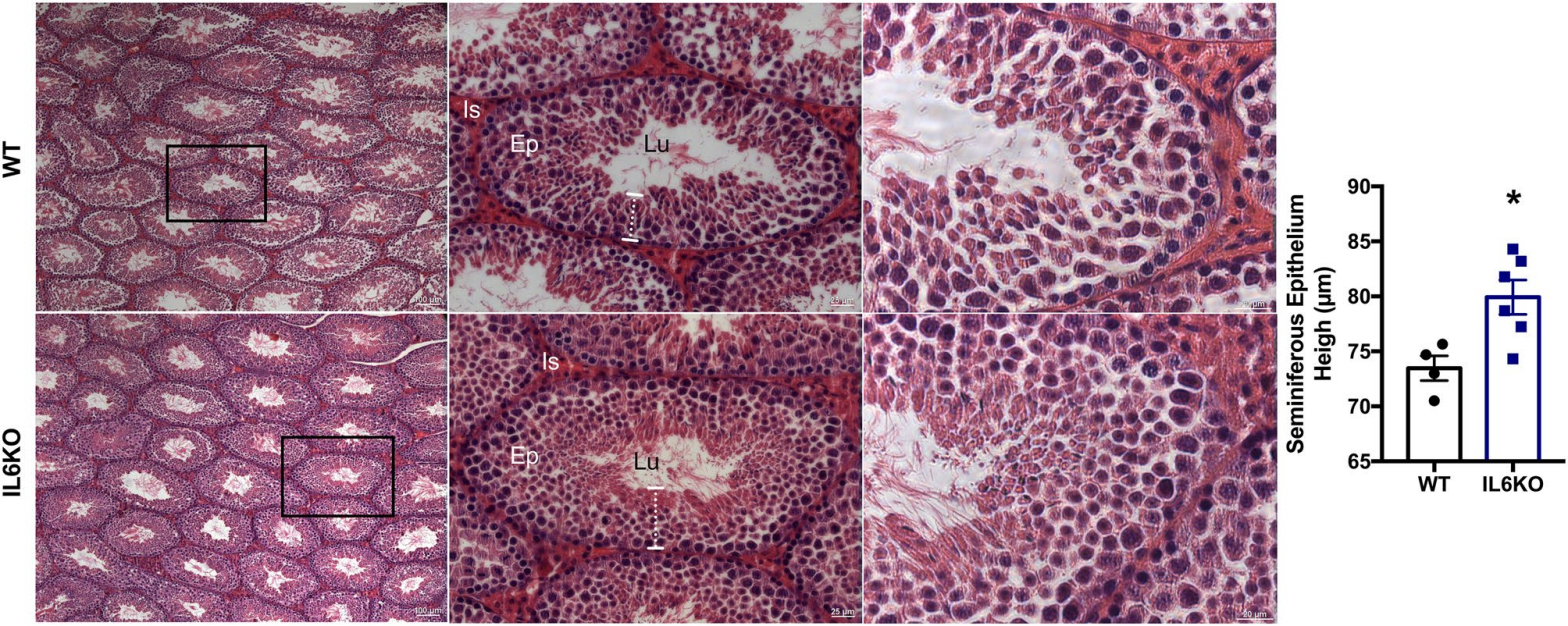
Testicular morphometric and histological analyses: WT group (**a**) and IL6KO group (**b**). Thicker layer of the seminiferous epithelium in the testes of IL6KO mice (*p* = 0.0381) without apparent morphological alteration. *p < 0.05, Mann–Whitney test. Values expressed as mean ± SEM. Results are representative of samples from 4 to 6 mice per group. The images show the seminiferous tubule under obj.5x, obj.20 × and obj.40× magnifications (from left to right, respectively). Ep, seminiferous epithelium; Lu, lumen; Is, interstitial space. Black rectangle (left panel) shows the seminiferous tubule in the middle panel under high magnification. White dotted line (middle panel) indicates the seminiferous epithelium thickness.

The absence of *Il6* increased the DSP (*p* = 0.0260) and the number of testicular spermatids (*p* = 0.0260), but it did not change the amount of sperm nor the transit time at the epididymal level (Table 2). Therefore, we further evaluated the testicular histological images, and although no morphological changes were observed in any of the groups, the height of the seminiferous epithelium was higher on the IL6KO group (*p* = 0.0381) (Fig. 1). The results showed that the lack of *Il6* interfered with sperm production.

**Table 2:** Sperm Parameters (number and epididymal transit time).

	WT (n = 6)	IL6KO (n = 5)
Spermatid number (× 10^6^/testis)	16.65 ± 1.39	21.07 ± 1.17 *
Daily sperm production (× 10^6^/testis/day)	3.44 ± 0.29	4.35 ± 0.24 *
Caput/corpus epididymis sperm number (× 10^6^/organ)	13.09 ± 0.49	15.98 ± 2.02
Cauda epididymis sperm number (× 10^6^/organ)	15.37 ± 1.81	17.87 ± 1.99
Caput/corpus epididymis sperm transit time (days)	3.96 ± 0.40	3.73 ± 0.55
Cauda epididymis sperm transit time (days)	4.83 ± 0.94	4.03 ± 0.28

*p < 0.05, unpaired *t*-test or Mann–Whitney test, GraphPad Prism 6. Values expressed as mean ± SEM.

#### Increased testicular testosterone and dihydrotestosterone levels due to *Il6* absence

**Figure 2:**
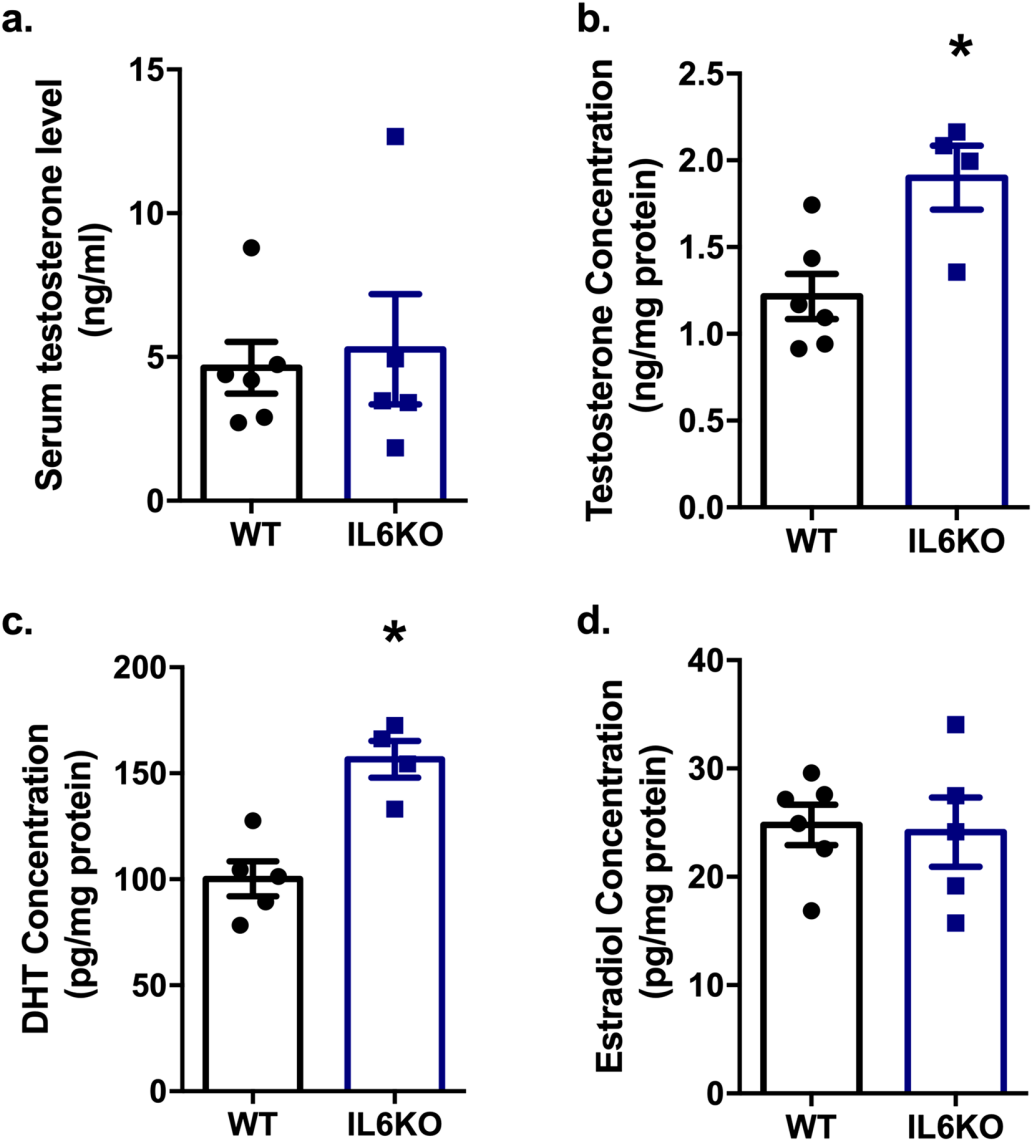
Effects of *Il6* absence on sex hormone levels: Circulating serum testosterone level (**a**); and testicular testosterone (**b**), DHT (**c**), and estradiol **(d)** concentration. Increased testosterone (p = 0.0381) and DHT (*p* = 0.0159) testicular levels in IL6KO mice. *p < 0.05, unpaired t-test or Mann–Whitney test. Values expressed as mean ± SEM. Results are representative of samples from 4 to 6 mice per group. DHT—dihydrotestosterone.

Despite serum testosterone remained unchanged (Fig. 2a), testicular testosterone (*p* = 0.0381) (Fig. 2b) and dihydrotestosterone (DHT) (*p* = 0.0159) (Fig. 2c) levels were increased on IL6KO mice, with no changes on estradiol (Fig. 2d) levels. The results showed that the change on sperm production is accompanied by testicular hormonal changes.

### The absence of *Il6* interfered with testicular JAK-STAT signaling pathway

**Figure 3:**
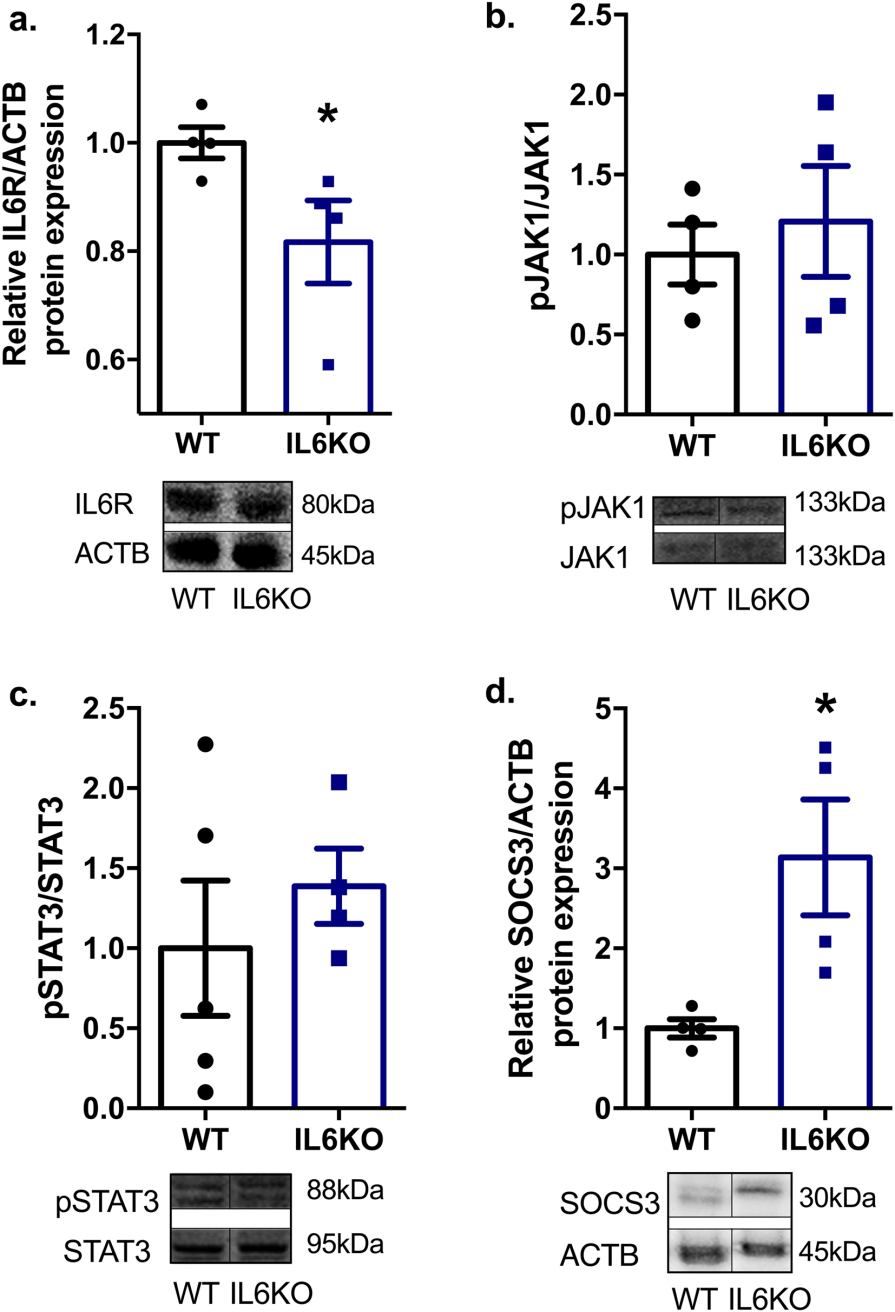
Testicular protein expression of IL6 signaling pathway components via JAK-STAT in IL6KO mice: expression of IL6R (**a**); pJAK1/JAK1 (**b**), pSTAT3/STAT3 (**c**) and SOCS3 **(d)** normalized by ACTB, and their respective representative western blot images. Lack of *Il6* decreases IL6R (*p* = 0.0286) and enhances SOCS3 (*p* = 0.0286) expression with no changes on JAK1-STAT3 expression. Full-length blots are presented in Supplementary Fig. 2. *p < 0.05, Mann–Whitney test. Values expressed as mean ± SEM. Results are representative of samples from 4 to 6 mice per group. ACTB – beta-actin; IL6R—interleukin-6 receptor, alpha; JAK1—Janus kinase 1; pJAK1—phosphorylated JAK1; STAT3—signal transducer and activator transcription 3; pSTAT3—phosphorylated STAT3; SOCS3—suppressor of cytokine signaling 3.

In order to determine whether *Il6* deficiency interferes with the JAK-STAT signaling pathway in the testis, and to investigate possible molecular mechanisms involved in the increase of steroidogenesis and subsequently augmented spermatogenesis, we analyzed the expression of the following proteins by western blotting: IL6R, JAK1, STAT3, and SOCS3. The absence of *Il6* reduced IL6R expression (*p* = 0.0286) (Fig. 3a), probably because of the lack of ligand. Moreover, it increased SOCS3 expression (*p* = 0.0286) (Fig. 3d) with no changes on JAK1 (Fig. 3b) and STAT3 (Fig. 3c) activation, which is demonstrated by the phosphorylated-total protein ratio. The results showed that the loss of *Il6* interfered with the components of testicular IL6-JAK-STAT signaling pathway and suggested that SOCS3 may be related to sex hormones and sperm production.

## Discussion

The results of this study indicate that IL6KO mice exhibit increased sperm production, testicular spermatids, seminiferous epithelium height, relative testis weight, and testicular testosterone and DHT levels, with no changes in sperm transit time and serum testosterone. We also found that IL6KO animals are leaner than WT animals, with reduced epididymal adipose tissue, as previously demonstrated by Fäldt et al.^34^.

*Il6* deficiency did not affect the absolute weight of the tissues analyzed, except for epididymal adipose tissue. Although the animals used in this study were not exactly the same age as the animals used in the study by Fäldt et al*.*^34^, they showed that 4-month old IL6KO mice are leaner than WT mice, mainly due to reduced fat mass, but there are no differences regarding free fat mass, which is in line with our results.

Regarding the testes and spermatogenesis in IL6KO mice, the loss of *Il6* interferes with testicular function. They showed increased daily sperm production and testicular spermatids, which is also observed by the increased height of seminiferous epithelium. Notwithstanding, the influence on sperm production, sperm number and sperm transit time in epididymal portions did not differ from WT mice. However, we noticed a trend towards increased relative epididymis weight. These data suggest that some spermatids may be undergoing apoptosis before reaching the epididymis in IL6KO mice. Thus, further studies are necessary to elucidate this assumption and whether *Il6* absence alters the epididymal morphology and function.

Spermatogenesis is the main role of testes, but the production of steroid hormones is the main secondary function of testicles and is essential for germ cell production. High IL6 levels compromise steroidogenesis, suppressing testosterone secretion by Leydig cells ^32^. Increased IL6 levels during murine chronic cysticercosis induce a feminization phenotype. However, this phenotype does not occur in IL6KO animals ^17^.

Additionally, IL6 up-regulates testicular aromatase activity^17^, which increases the conversion of testosterone into estradiol. The lack of *Il6* led to higher testicular testosterone and DHT levels, with no changes in testicular estradiol and serum testosterone levels. These data suggest an androgenic stimulation that could be involved in the increase of spermatogenesis.

Proteins such as STAT3 and SOCS3 are related to both IL6 signaling and testicular functions^14,19,35^. Thus, we analyzed whether *Il6*-null mice present any alteration on the testicular JAK-STAT signaling pathway, once changes on other tissues of IL6KO mice have already been found^36^. Interestingly, although there was no change on pJAK1/JAK1 and pSTAT3/STAT3 ratios, the protein content of SOCS3 was shown to be increased. Sarvas et al.^36^ observed a five-fold increase of SOCS3 in the liver of IL6KO mice, with no differences regarding leptin or pAKT. Most of the literature concerning SOCS3 and testes is related to inflammation, and high levels of SOCS3 are often associated with a reduction of STAT3 phosphorylation, and subsequent spermatogenesis impairment^35,37^, testicular atrophy^35^, and serum testosterone reduction ^35^. The results found in the present study do not match this scenario.

Moreover, the JAK-STAT pathway is not the only pathway to up-regulate SOCS3, which can also occur via transcription factor cAMP responsive element binding protein (CREB), for instance^38^. Chakrabarti et al. and Kim et al.^39,40^ have already shown it in microglia and stromal cells; however, to date, no studies have related the expression of SOCS3 in testis with the cAMP-dependent pathway. Nonetheless, the participation of CREB during spermatogenesis and steroidogenesis has already been proved^41,42^. Besides that, STAT3 is not the only target of SOCS3^43,44^. Furthermore, it has already been demonstrated that the lack of SOCS3 in steroidogenic factor-1 cells results in small testes, with no changes in serum testosterone levels and fertility^45^. Therefore, our hypothesis is that the increase of SOCS3 stimulates testicular androgen production, possibly via CREB, which results in increased testicular relative weight and spermatogenesis in the absence of *Il6*.

**Figure 4:**
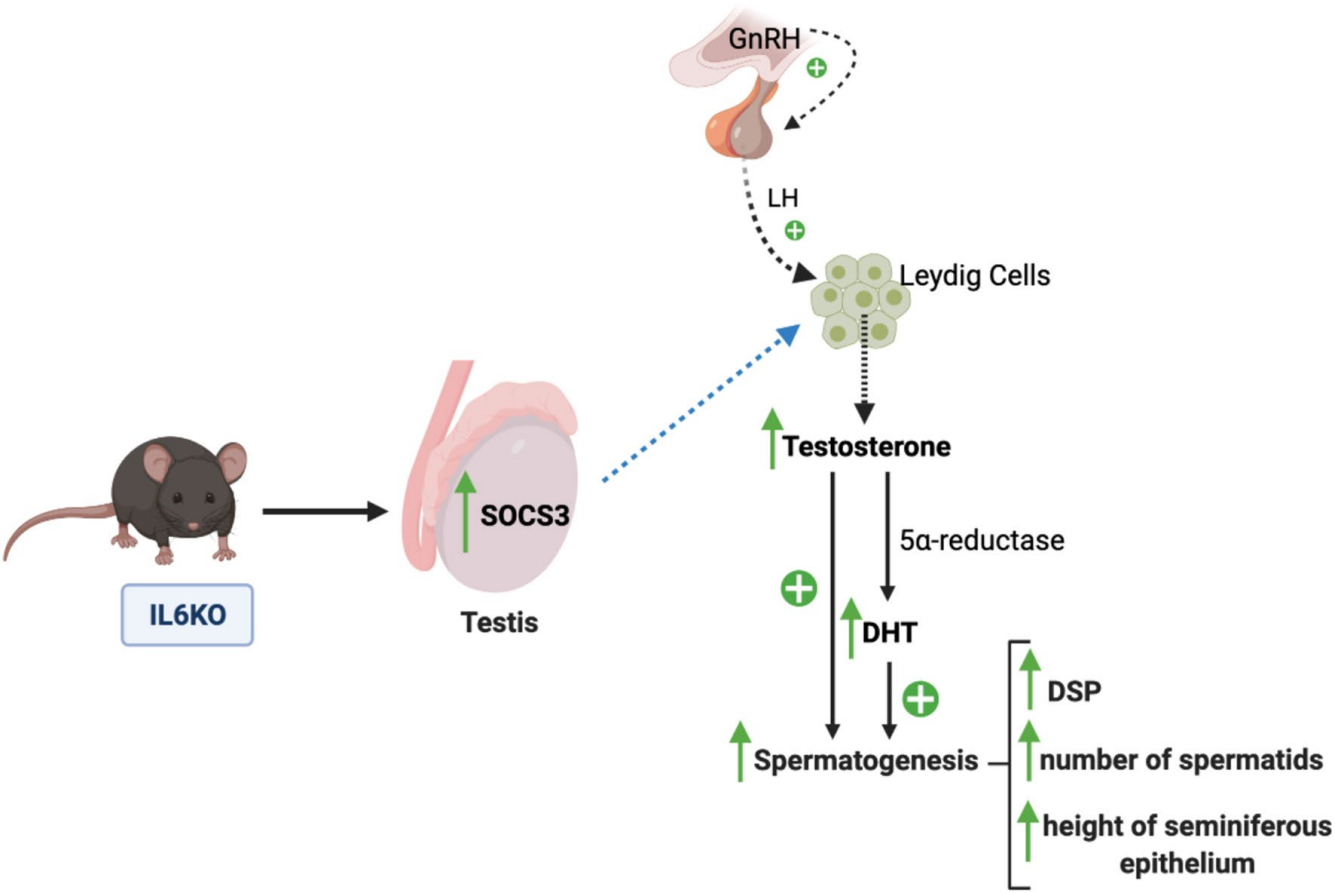
Summary illustration showing the effects of *Il6* absence on the testis. The GnRH produced by the hypothalamus stimulates LH secretion by the pituitary, which triggers the testosterone production by Leydig cells in the testicles. In IL6KO mice, we observed an increased testicular SOCS3 expression. We suggest that the increase of SOCS3 leads to an increased production of testosterone, which is converted by the 5⍺-reductase enzyme into its bioactive metabolite (DHT). Enhanced testicular androgens increase spermatogenesis, which is noted by the increased daily sperm production (DSP), leading to the increased number of spermatids and the thicker layer of the seminiferous epithelium. GnRH—gonadotropin-releasing hormone; LH—luteinizing hormone; DHT—dihydrotestosterone; DSP—daily sperm production; SOCS3—suppressor of cytokine signaling 3. Blue dashed arrow indicates a suggestion.

Given the above, we conclude that the loss of *Il6* interferes with testicular function, by increasing sperm and androgen production. Increased daily sperm production leads to a higher spermatid number and increased height of the seminiferous epithelium, which occurs due to the stimulation by the testicular androgens. We suggest that the mechanism by which *Il6* deficiency drives androgenic production is the increase of SOCS3 in the testis (Fig. 4), which occurs independently from the JAK-STAT pathway. Further investigation is required to confirm this assumption and to evaluate the effects in the epididymis.

## Methods

### Animals

Twenty-week old male C57Bl/6J WT (n = 15) and B6.129S2-*Il6*^*tm1Kopf*^/J (IL6KO) (JAX stock #002650) (n = 12) mice from Jackson Laboratory were used in this study. The animals were housed in a room with 12-h artificial illumination (06:00 a.m.–06:00 p.m.) and controlled temperature (22 ± 2 °C). Food and water were given ad libitum. All animal testing protocols were performed according to the Brazilian National Council of Animal Experiment Control (CONCEA) and approved by the Animal Use Ethics Committee (CEUA UNIFESP Permit Number: 1990020316) and the Internal Biosafety Committee (CIBio UNIFESP Permit Number: 2016/10) of the Federal University of São Paulo, following the ARRIVE guidelines.

In order to ensure that the IL6KO group had no functional IL6, three animals per group were challenged with LPS (which has previously been shown to elevate serum and testicular IL6), and the lack of functional protein in these animals was confirmed by ELISA^22^ (Supplementary Fig. 1). All other experiments were carried out in non-injected mice.

### LPS Injection

Three mice from each genotype, WT and IL6KO, received a single dose of 5 mg/kg LPS (*Escherichia coli* O111:B4—L2630—Sigma-Aldrich, EUA) via intraperitoneal injection. The animals were euthanized 24 h after the administration of LPS. The serum and testes were collected and kept frozen at -80 °C until IL6 testing by ELISA.

### Material collection

The animals were euthanized by cervical dislocation after anesthesia with 30% xylazine (10 mg/kg, intraperitoneal) and ketamine (150 mg/kg, intraperitoneal) solution. The testes, whole epididymis, epididymal adipose tissue, muscle (gastrocnemius), and liver were removed and weighed. The whole blood was collected by cardiac puncture to measure sex hormones.

### Sperm count and epididymal transit time

Homogenization-resistant spermatids (step 14–16 of spermatogenesis) from both testes and homogenization-resistant sperm from both caput/corpus and cauda epididymis were counted, as previously described by Robb *et. al*^46^, Meistrich^47^, and Turgut *et al*.^48^, with some adaptations, as follows: after the removal of tunica albuginea from testes, they were homogenized in 1 ml ST solution (0.9% NaCl, 0.5% Triton X-100), followed by sonication at 80 mA for 30 s.

The homogenates were diluted 1:10 in ST solution, and a small sample was transferred into the Neubauer chamber (4 fields per animal) for counting elongated spermatids. The daily sperm production (DSP) was then calculated by dividing the number of elongated spermatids by 4.84 (the number of days spermatids spend in stage 14–16 during murine spermatogenesis)^49^, after dilution factor correction.

For sperm count in the epididymis portions, small cuts were performed in caput/corpus and cauda epididymis in order to facilitate the sperm release, and then homogenized and counted as described above. Epididymal transit time was calculated by dividing the number of sperms within each epididymal region by DSP^46,50^.

### Histological and morphometric analysis

Histological and morphometric analyses were blindly performed on testicular 5 µm paraffin cross-sections stained with hematoxylin and eosin (H&E), after fixation in 4% paraformaldehyde (4% PFA) and 70% ethanol. The height of the seminiferous epithelium was measured through observation with a 10× objective lens under optical light microscopy using Image J software. The measure was obtained from the average of four measurements per tubule with three slices per animal.

### Protein extraction and western blotting

The homogenization process for protein extraction was performed with whole testis in 800 µl of lysis buffer composed by 100 mM Tris–HCl (pH 7.5), 1% Triton X-100, 10 mM EDTA, 100 mM sodium fluorite, 10 mM sodium pyrophosphate, 10 mM sodium orthovanadate, 2 mM phenylmethylsulphonyl fluorite (PMSF), and 0.1 mg aprotinin/mL using a Polytron homogenizer. The homogenate was centrifuged at 14,000 rpm for 40 min at 4ºC. The supernatant was kept on ice, and the total protein concentration was measured by the Bradford method (Bio-Rad Laboratories, Inc.) in a plate spectrophotometer. The samples were submitted to 10% SDS–polyacrylamide gel electrophoresis and transferred into nitrocellulose membrane.

The membranes were blocked with 1% BSA for 2 h at room temperature. The primary antibodies for IL6R (ab83053, Abcam), pJAK1 (ab138005, Abcam), JAK1 (ab47435, Abcam), pSTAT3 (ab76315, Abcam), STAT3 (ab31370, Abcam) and SOCS3 (ab16030, Abcam) were used to detect the target protein at 1:1000 dilution, overnight at room temperature. Subsequently, the membranes were incubated with appropriate secondary antibodies: horseradish peroxidase-conjugated anti-rabbit and/or anti-rat IgG for 1 h. In order to quantify the densities of the bands obtained from the chemiluminescent membrane, the Scion Image Software for Windows was used as arbitrary unit. Target protein normalization was performed with the housekeeping beta-actin (ACTB, 1:5000; Cell Signaling).

### Enzyme-linked immunosorbent assay (ELISA)

Serum testosterone and testicular testosterone, dihydrotestosterone (DHT), and estradiol levels were measured by IBL-America ELISA kits. The serum and testicular IL6 levels after LPS challenge were measured by Mouse IL6 Quantikine ELISA kit M6000B. The analyses were performed following the manufacturer’s instructions (Manufacturer: IBL-America and R&D Systems). Testicular protein extraction was performed as previously described in the protein extraction for western blotting.

### Statistical analysis

The quantitative values were presented as mean ± standard error of the mean (SEM). The difference between the groups was evaluated by Student’s unpaired t-test or non-parametrical Mann–Whitney test, depending on the data normality distribution. The data normality was evaluated by the Kolmogorov–Smirnov test—if *p*-value > 0.05, the data are treated as parametrical variables. Statistical significance was assigned at *p* < 0.05. Statistical analysis was performed by using GraphPad Prism Software version 6.0.

## Supplementary Information

Supplementary Information.
