# Supplementary material for: Interleukin-6 deficiency modulates testicular function by increasing the expression of suppressor of cytokine signaling 3 (SOCS3) in mice
Source: Sci Rep. 2021 Jun 1;11:11456. doi: 10.1038/s41598-021-90872-6 (PMC8169872; doi:10.1038/s41598-021-90872-6)
Supplement: Supplementary file 1 — Supplementary Information. [file 41598_2021_90872_MOESM1_ESM.pdf]

Interleukin-6 deficiency modulates testicular function by increasing the expression of suppressor of cytokine signaling 3 (SOCS3) in mice

Thaís Alves-Silva[1,2], Geanne Arantes Freitas[3,4], Talita Guerreiro Rodrigues Húngaro[1,5], Adriano Cleis Arruda[1,5], Lila Missae Oyama[6], Maria Christina Werneck Avellar[3], Ronaldo Carvalho Araujo[1,2,5]*

Authors affiliations:

[1]Laboratory of Genetics and Exercise Metabolism, Biophysics Department, Federal University of São Paulo (UNIFESP), Brazil;

[2]Molecular Biology Program, Federal University of São Paulo (UNIFESP), Brazil;

[3]Pharmacology and Molecular Biology Institute, Federal University of São Paulo (UNIFESP), Brazil;

[4]Institute of Biomedical Sciences, University of São Paulo (USP), Brazil;

[5]Nephrology Program, Federal University of São Paulo (UNIFESP), Brazil;

[6]Laboratory of Nutrition and Endocrine Physiology, Physiology Department, Federal University of São Paulo (UNIFESP), Brazil.

*corresponding author: araujorona@gmail.com

Supplementary Result

Supplementary Result 1: The IL6KO group does not have functional IL6

The IL6KO mice have no increase of IL6 after LPS challenge (Supplementary Fig. 1), as observed in WT group, which validates the deficiency of *Il6* in this group.

Supplementary Figures

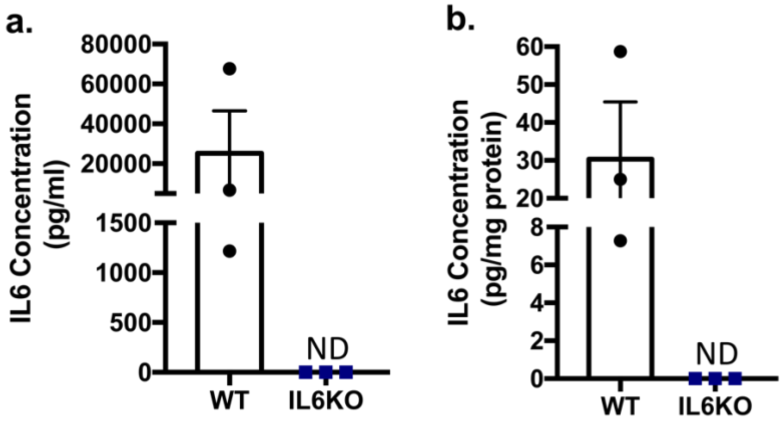

Supplementary Figure 1. IL6 concentration after LPS challenge (5mg/kg, single injection, i.p.) in mice: serum (**a**) and testicular (**b**) levels. The animals were euthanized 24 hours after the LPS administration. Values expressed as mean ± SEM; n=3 per group. ND: no detected.

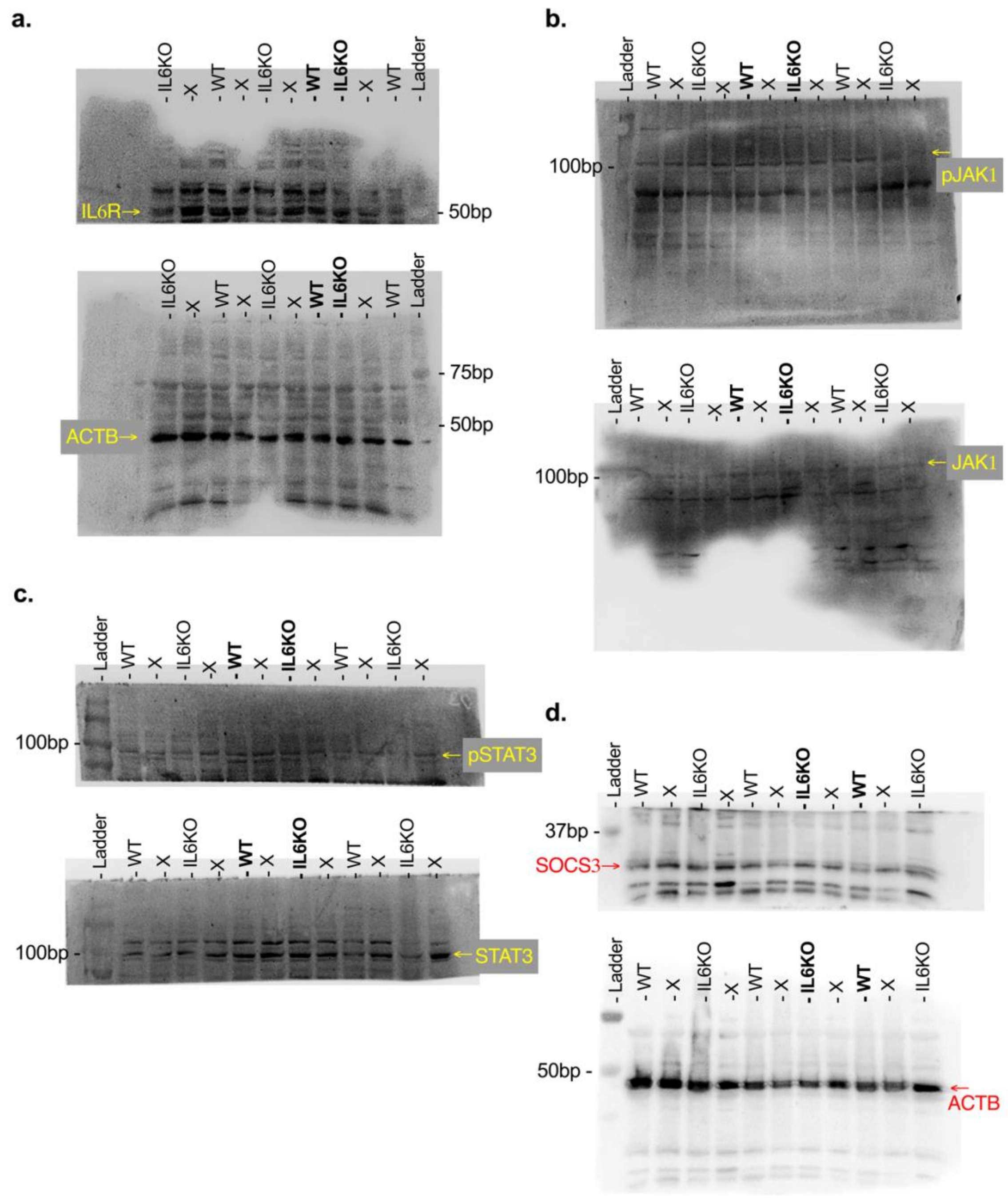

Supplementary Figure 2. Testicular protein expression of IL6 signaling pathway components via JAK-STAT in IL6KO mice: Expression of IL6R (**a**); pJAK1/JAK1 (**b**), pSTAT3/STAT3 (**c**) and SOCS3 **(d)** normalized by ACTB, and their respective representative western blot images. Lack of *Il6* decreases IL6R (p=0.0286) and enhances SOCS3 (p=0.0286) expression with no changes on JAK1-STAT3 expression (Figure 3). Representative bands are identified in bold. X – samples not used in this manuscript. ACTB – beta-actin; IL6R – interleukin-6 receptor, alpha; JAK1 – Janus kinase 1; pJAK1 – phosphorylated JAK1; STAT3 – signal transducer and activator transcription 3; pSTAT3 – phosphorylated STAT3; SOCS3 – suppressor of cytokine signaling 3.
